# Radiation exposure to staff during fluoroscopic endoscopic procedures

**DOI:** 10.1002/deo2.234

**Published:** 2023-04-05

**Authors:** Mohamed Khaldoun Badawy, Ellen Henely‐Smith, Solaiman Hasmat

**Affiliations:** ^1^ Monash Health Imaging Monash Health Clayton Australia; ^2^ Department of Medical Imaging and Radiation Sciences, School of Primary and Allied Health Care, Faculty of Medicine, Nursing and Health Sciences Monash University Clayton Australia

**Keywords:** endoscopy, fluoroscopy, ionizing radiation, radiation exposure, radiation safety

## Abstract

**Objectives:**

Fluoroscopically guided procedures utilize ionizing radiation to assist in the diagnosis and treatment of the patient. The use of ionizing radiation is not without risk to the operator and other staff members present during endoscopic procedures. This study simulates radiation exposure during endoscopic retrograde cholangiopancreatography procedures under different shielded conditions and provides practical radiation safety recommendations, through easy‐to‐use visual guides.

**Methods:**

We obtained radiation exposure measurements at varying locations with different shielding setups surrounding a mobile C‐arm fluoroscopic unit while imaging a patient equivalent phantom at different heights. Heat maps were generated for the various conditions to provide visual guides for radiation protection.

**Results:**

Different heat maps detailing various shielding methods have been generated to assist in determining the dose rate at varying locations surrounding the patient. The use of appropriate radiation protection could decrease the staff dose by up to 98%.

**Conclusion:**

Although minor per procedure, the magnitude of radiation exposure will accumulate over the staff's working life. As such, it is recommended that precautions be taken during fluoroscopically guided endoscopy procedures to ensure radiation is kept as low as reasonably achievable.

## INTRODUCTION

Staff radiation safety needs constant revision to ensure occupational exposure remains low with the growing utility of fluoroscopic equipment. Endoscopic retrograde cholangiopancreatography (ERCP) is a subset of endoscopic procedures used to diagnose and treat the liver, gallbladder, bile ducts, and pancreas. When ERCP procedures are fluoroscopically guided, the abdomen region is exposed to ionizing radiation. Due to the density of the abdominal area, a large amount of radiation is scattered from the patient, exposing staff within the room. Although radiation dose to staff is relatively low per individual procedure, the high rate of endoscopic procedures involving fluoroscopic equipment can accumulate staff radiation dose over time.

Endoscopists are exposed to a relatively low dose to the body's trunk if appropriate personal protective equipment (PPE), such as lead shielding from shoulders to knees with a thyroid collar. However, the eye and hand dose was a concern.^1^, ^2^, ^3^, ^4^, ^5^ Multiple studies indicate that radioprotective eyewear should be a part of the standard PPE due to the high chance of exceeding the lens dose occupational limit.^1^, ^5^, ^6^, ^7^ A further study indicated that compliance with the standard PPE or lead gown and thyroid collar might be a concern, with 27% of endoscopists responding to not wearing thyroid collars and only 10% wearing a radiation monitoring badge.^8^

Although several studies report radiation doses to the staff during ERCP procedures, it is difficult to gauge the dose for unmonitored staff. Furthermore, there is limited information on mobile lead shields and the radiation dose reduction for other staff within the shield region. This phantom study aims to identify practical radiation safety for endoscopy staff during ERCP by measuring the scattered radiation at various positions in the procedural room and providing position‐specific advice. This advice will be given in easy‐to‐use visual guides to aid with implementation. Additionally, the usefulness of mobile shields in protecting staff beyond the primary user will be explored.

## MATERIALS AND METHODS

**FIGURE 1:**
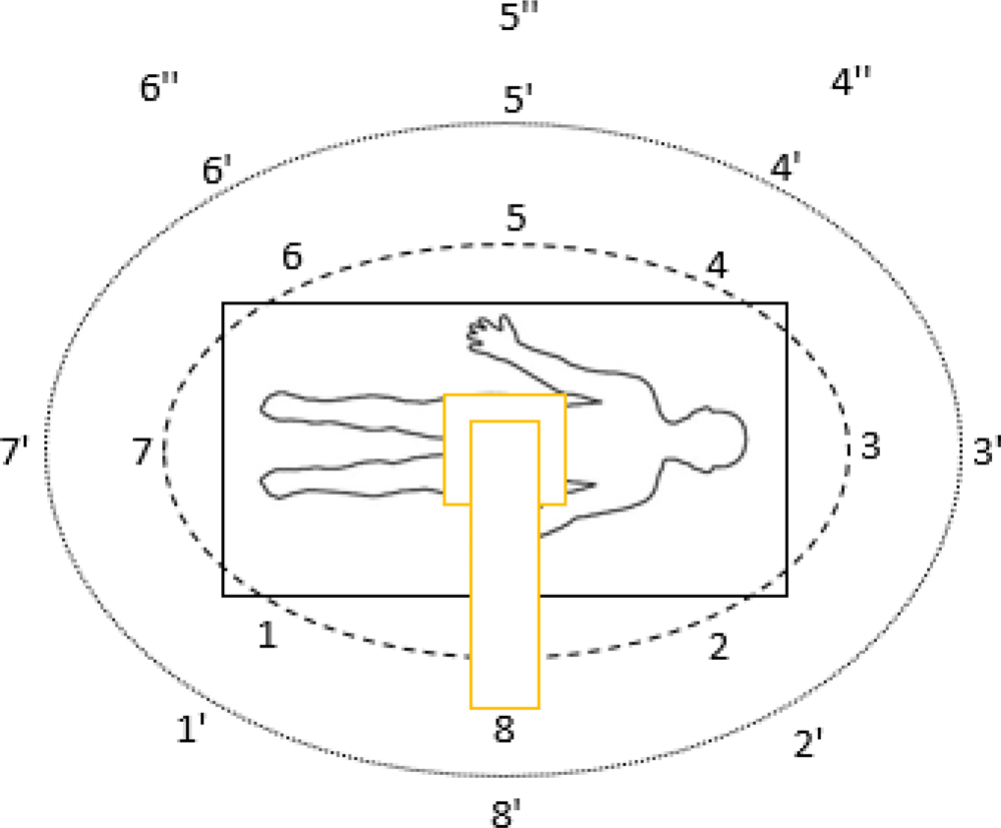
Experimental setup and positions used to measure the scatter radiation.

A Phillips BV Endura Image Intensifier was used over a radiolucent bed and a 25 cm polymethylmethacrylate (PMMA) phantom to replicate an adult abdomen. The personal dose equivalent, H_p_(10), was recorded at various locations (Figure 1) around the patient bed on a PTW STEP OD‐02 survey meter (PTW Freiburg GmbH, Freiburg, Germany) at the height of 1.3m in µSv/h to estimate whole body dose. Measurements at heights of 1.2 m were taken without the cap in H_p_(0.07) mode, measured in µSv/h to simulate the dose to the hands. The measurements at the height of 1.65 m were converted to H_p_(3) as an approximation for eye dose using the conversion factors presented in International Commission on Radiation Units and Measurements Report 95.^9^

Measurements were taken at positions 1–8 at a 1.0 m distance from the center of the PMMA and heights of 1.3 and 1.65 m to approximate the whole‐body effective dose and dose to the lens of the eye, respectively. (Figure 1). The PMMA was exposed at 71 kVp, and 2.3 mA, and the measurements were taken for 10 s to allow time for the dose rate to stabilize. The exposures were then repeated at positions 1’–8’ at a distance of 1.5m from the center of the PMMA at heights of 1.2 and 1.65 m to interpolate the data. Additional measurements were taken at positions 4“–6” at 2 m from the center of the PMMA. All Measurement at each location was repeated three times, and the mean was calculated.

A 2.5 mm lead equivalent lead shield was placed in front of positions 4, 5, and 6, and measurements were taken at 1.0, 1.5, and 2.0 m from the center of the PMMA. Measurements were repeated with a radioprotective gown of 0.5 mm lead equivalence between the patient and positions 4, 5, and 6. Interpolation from the data measured at 1, 1.5, and 2 m was used to determine dose rates at additional positions to create a heat map as a visual representation, with and without lead shielding

Finally, measurements were recorded at the location where the gastroenterologist would be situated during a procedure, 0.5 m away from the center of the PMMA, at heights of 1.2, 1.3, and 1.65 m to approximate the dose at the hands, abdomen, and eyes of the primary operator.

Data pertaining to exposure parameters (kVp and mA) and fluoroscopy times were mined from the Radiology Information System for ERCP procedures conducted between June and December 2020. Studies with no fluoroscopic time or no units recorded were excluded from the data set. The interpolation, median, and interquartile range (IQR) were calculated in Microsoft Excel for Mac V16.56. Using conditional formatting, the measured and interpolated data was used to create easy‐to‐use heat maps as visual guides in Microsoft Excel.

## RESULTS

**FIGURE 2:**
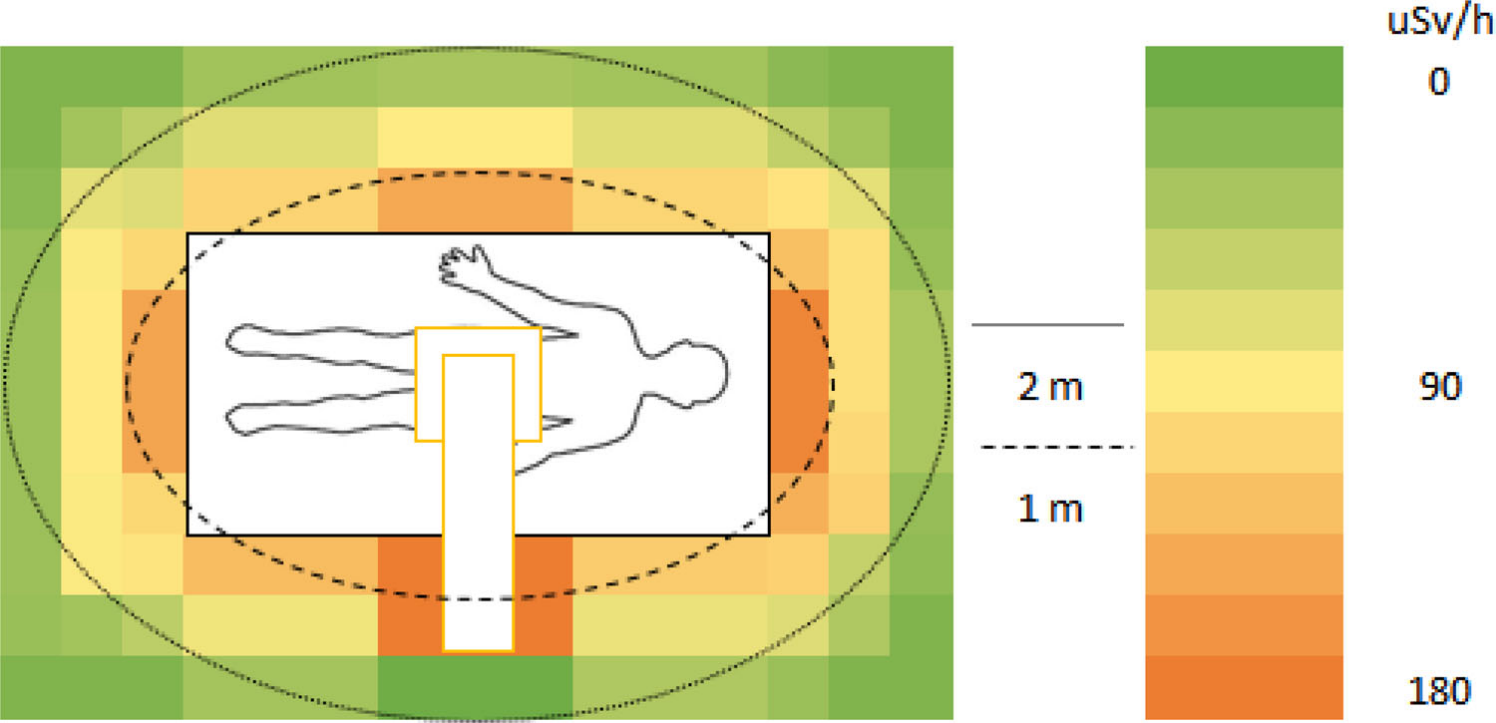
Approximated unshielded personal dose equivalent rate H_p_(10) during a simulated endoscopic retrograde cholangiopancreatography procedure.

**FIGURE 3:**
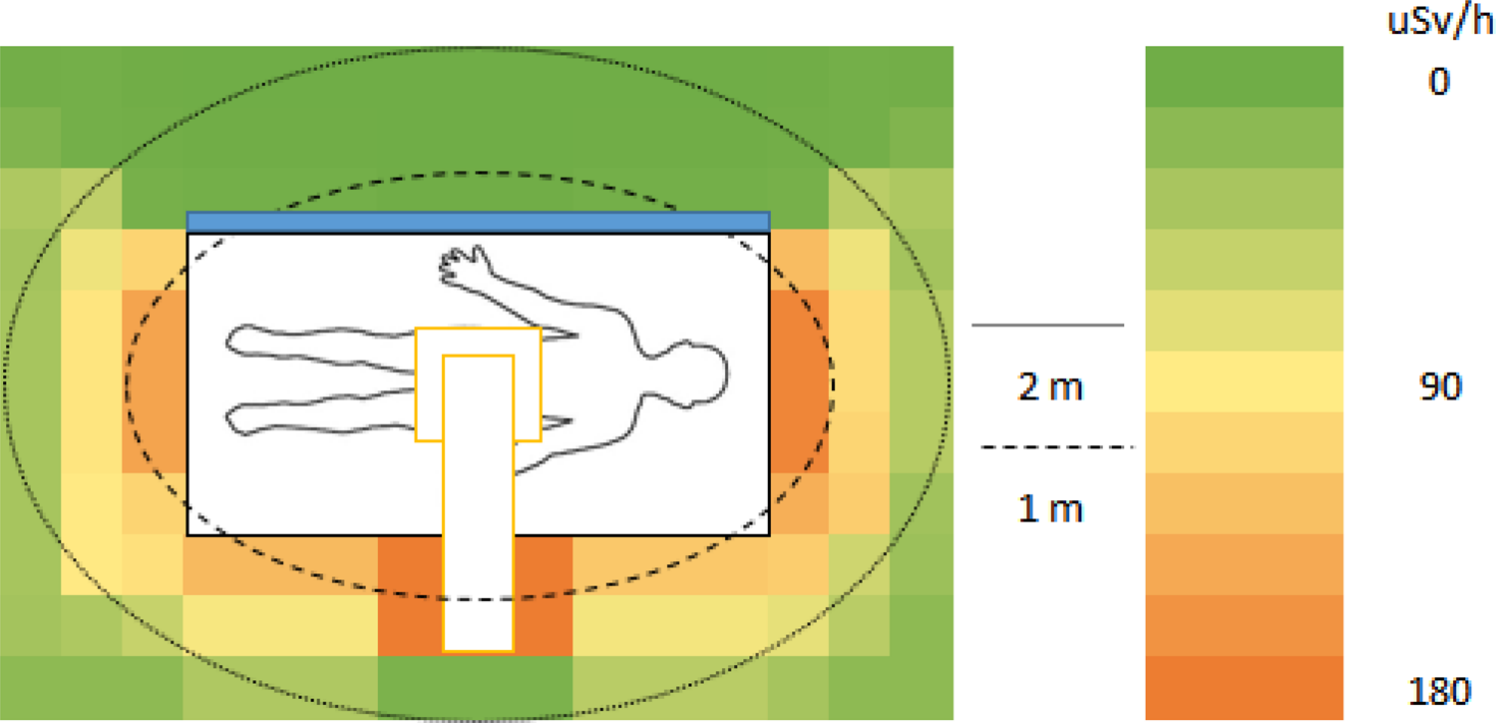
Approximated personal dose equivalent rate Hp(10) during a simulated endoscopic retrograde cholangiopancreatography procedure using a mobile lead shield between the operator and the patient.

**FIGURE 4:**
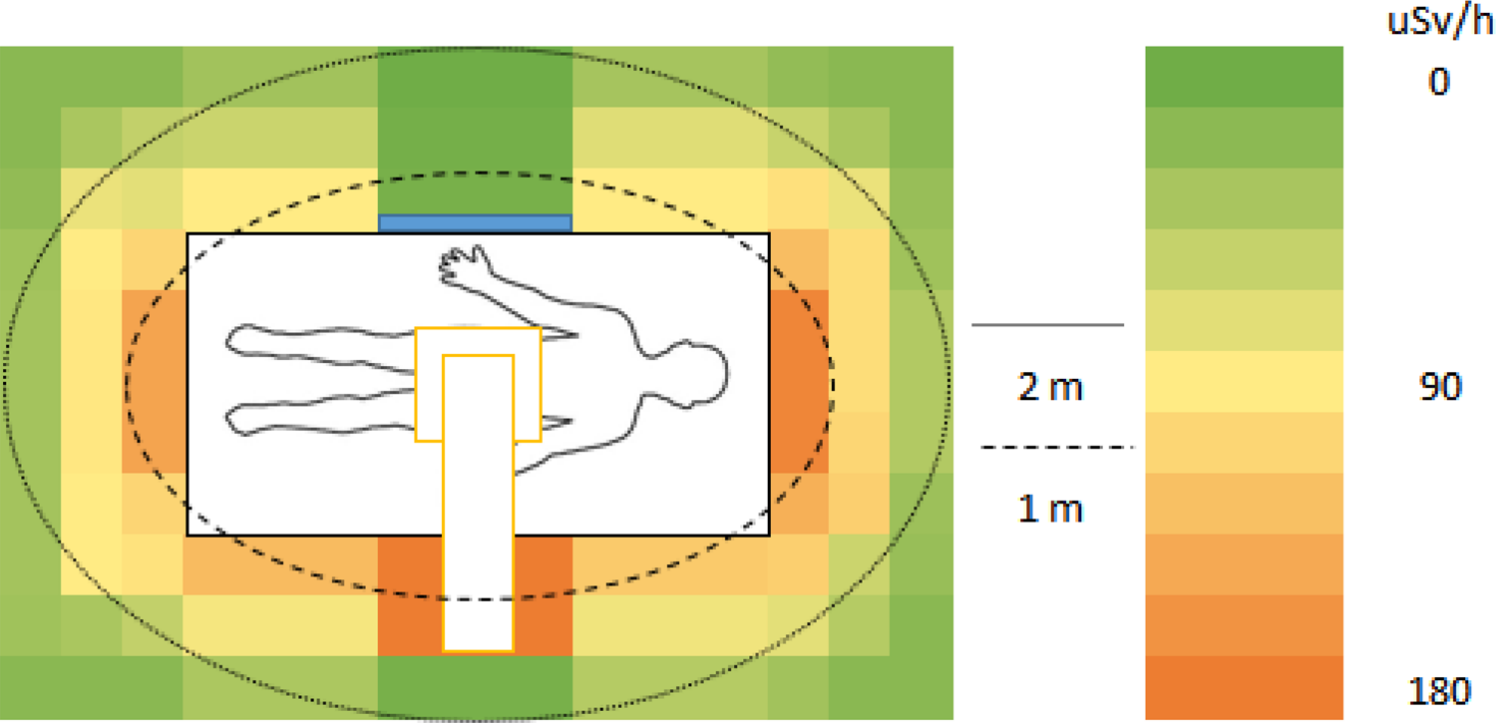
Approximated personal dose equivalent rate Hp(10) during a simulated endoscopic retrograde cholangiopancreatography procedure using a radioprotective apron between the operator and the patient.

In a simulated ERCP procedure, the dose rate was measured in H_p_(10) to approximate the unshielded whole‐body effective dose rate surrounding the PMMA phantom. The unshielded dose rate ranged between 112 and 173 µSv/h at 1 m and 9 and 47 µSv/h at 2 m (Figure 2). When a mobile shield was placed between the locations of the patient and the primary operator, the dose rate ranged between 2 and 173 µSv/h at 1 m and 1 and 47 µSv/h at 2 m (Figure 3). This resulted in a reduced dose rate of up to 98% in regions covered by the shield. When a lead apron was placed between the locations of the patient and the primary operator, the dose rate ranged between 9 and 173 µSv/h and 4 and 47 µSv/h at 2 m (Figure 4). This resulted in a dose rate reduction of up to 92% in the region behind the apron. The apron coverage and reduction in other areas were much smaller than the mobile shield.

The large variance in dose rate at different locations equidistant from the PMMA phantom is due to contributions from multiple sources. For example, locations closer to the C‐arm at 1 m had a higher dose rate potentially due to the contribution from scatter from the patient, tube leakage, and secondary scatter from the C‐arm itself. The maximum dose rate, 173 µSv/h, was measured behind the C‐arm location of the mobile fluoroscopic equipment.

**FIGURE 5:**
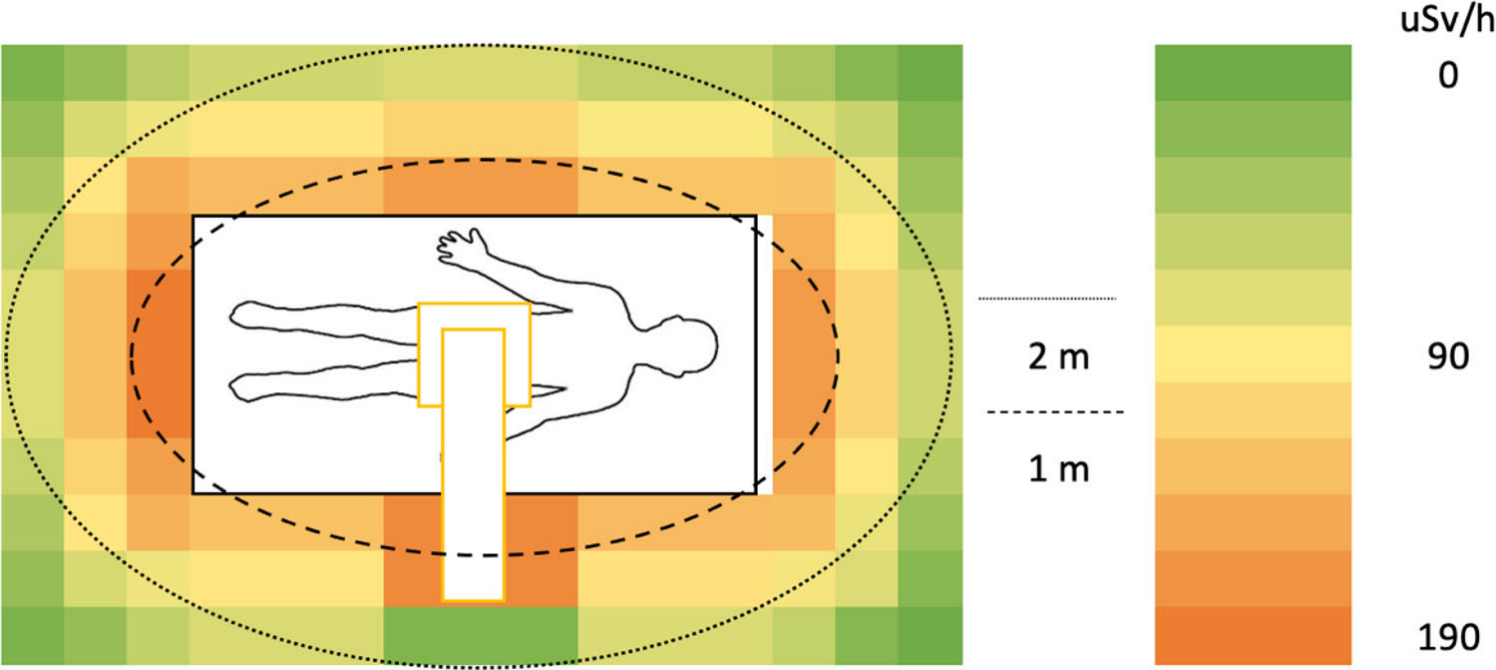
The dose rate to the lens of the eye, H_p_(3), approximated during simulated endoscopic retrograde cholangiopancreatography procedures.

The Hp(3) dose was calculated to approximate the eye lens dose rate in the simulated procedure. The unshielded lens dose rate was between 131 and 189 µSv/h at 1 m and 22 and 80 µSv/h at 2 m (Figure 5).

**FIGURE 6:**
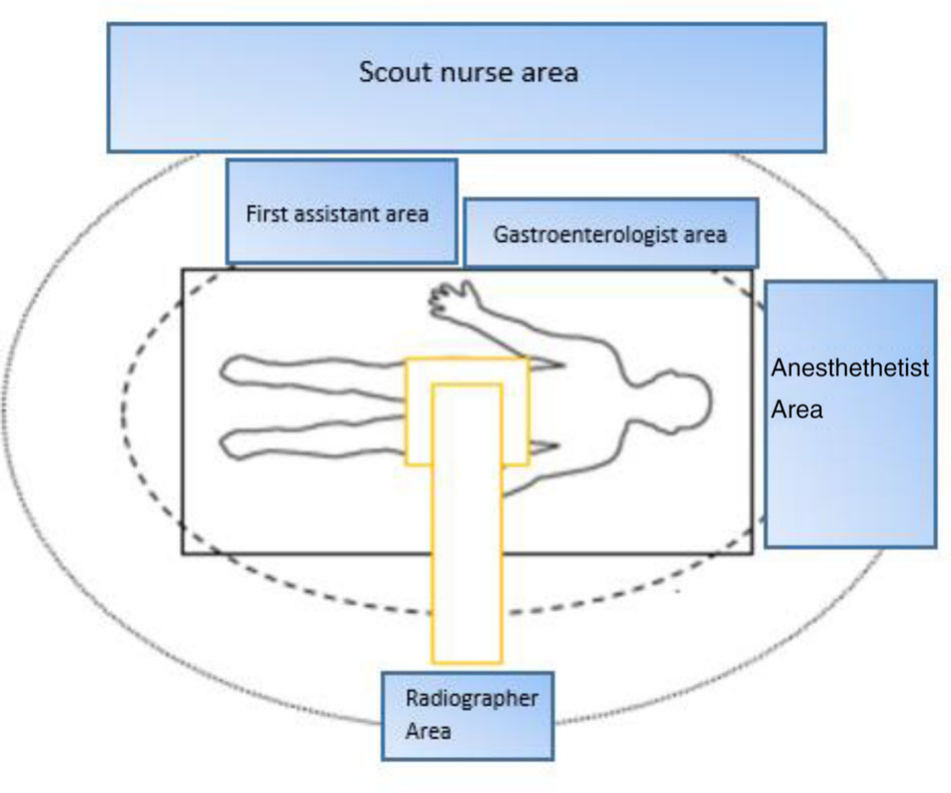
Typical staff locations within the endoscopy suite in our study setting.

**FIGURE 7:**
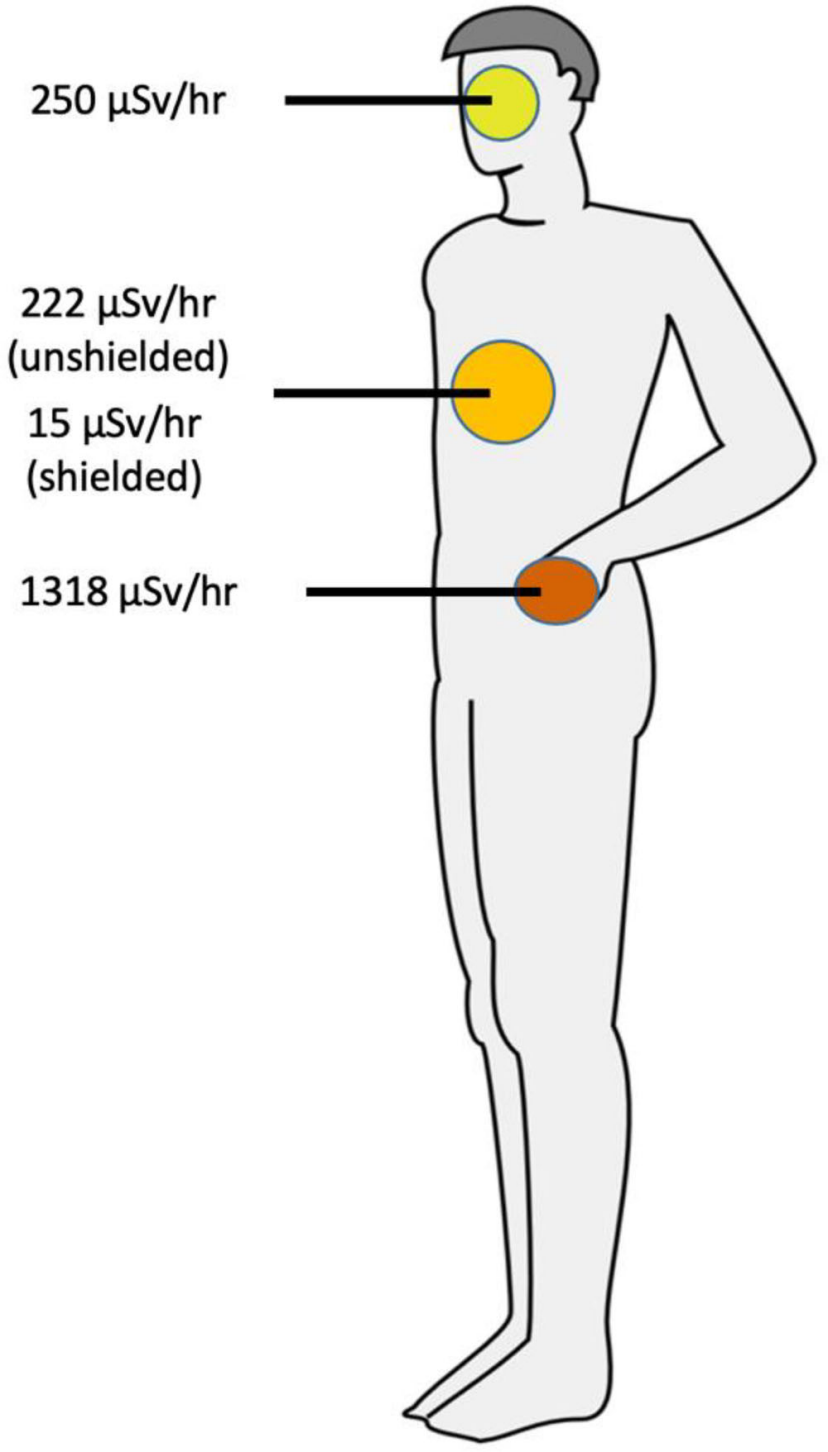
Radiation dose rate to the operator during a simulated endoscopic retrograde cholangiopancreatography (ERCP) procedure.

The dose rate was measured at the location where the gastroenterologist was situated (Figure 6) to approximate the whole body, lens, and finger dose. The maximum dose rate measured in H_p_(0.07) was found at the location of the hands and was 1318 µSv/h. The lens dose calculated in H_p_(3) was 250 µSv/h, and the personal dose equivalent measured in H_p_(10) was 15 µSv/h and 222 µSv/h for shielded and unshielded measurements, respectively (Figure 7).

In the 6 months investigated, there were 278 ERCP procedures conducted. Twenty‐six studies were excluded due to the fluoroscopy time or the units not being recorded. Overall, 252 studies were used to calculate the median fluoroscopy time. The studies in this sample recorded a median fluoroscopy time of 79 s (IQR = 44–141 s).

Table 1 reports the average personal dose equivalent and lens dose per procedure based on the median fluoroscopy time calculated. The location of the gastroenterologist was assumed based on local practice (Figure 6), and the average distances of 1 and 2 m are presented to allow for the estimation of the dose to other staff surrounding the patient.

**TABLE 1:** The estimated dose per procedure, µSv, at varying locations and distances based on the median fluoroscopy time of this study

	Gastroenterologist	1 m	2 m
Whole body (µSv)	3.3	3.8	1.0
The lens of the eye (µSv)	5.5	2.5	1.1
Hand (µSv)	28.9	–	–

## DISCUSSION

Our study provides a starting point for estimating the potential exposure to staff during fluoroscopic endoscopic procedures, This information can be used to develop strategies for minimizing the risk of radiation exposure in the clinical setting and may inform the use of protective measures, such as lead shielding, to reduce the dose received by staff members. Using this study's results as a dose rate can help scale the exposure to local practices by using the fluoroscopic time of a typical procedure.

The unshielded personal dose equivalent rate measured in multiple locations indicates that staff dose during an ERCP procedure may be substantial. Considering the approximated whole‐body effective dose of 3.3 µSv/procedure for a gastroenterologist. The same magnitude of exposure can be expected for any staff required to be closer than 1m from the patient. In these cases, exceeding 1 mSv without PPE is not unlikely, particularly considering that staff may be subjected to procedures or modalities that expose them to higher radiation doses during their work. Although staff at 2 m may be exposed to less radiation, it should still be a minimum requirement that all staff in the room during an ERCP procedure wear a radioprotective apron, including a thyroid collar, or stand behind a mobile shield. This is because controls must be introduced to ensure all workers who may exceed the public dose limits are protected and monitored to confirm that the occupational dose limit of 20 mSv/year is not exceeded.^10^ Additional controls, such as table‐suspended shields, have also been suggested for operators with a high workload with fluoroscopic equipment.^11^

The approximated radiation dose to the lens of the eye was 5.5 µSv/procedure for the gastroenterologist. A recent meta‐analysis found that the dose to the lens of the eye was even higher, 18 µSv/procedure for fixed effect analysis and 139 µSv/procedure for random effect analysis.^12^ Based on our results and those found in the literature,^11^, ^12^, ^13^, ^14^, ^15^ approaching or exceeding the annual dose constraint for the lens of the eye may be possible. Thus, the authors recommend that staff within 1m of the patient utilize radioprotective eyewear. A study also showed some promising results using radiation‐attenuation drapes placed on the patient to reduce unshielded exposure to the eyes; however, this type of drape is single‐use; thus, its cost‐effectiveness versus individual radioprotective eyewear should be considered.^16^ Staff typically located at distances greater than 1m are unlikely to approach the occupational limit. For this staff category, radioprotective eyewear should be optional as it may not provide a significant risk reduction.

The radiation dose measured at a location that approximated the hands of the gastroenterologist indicates that the hands could receive 28.9 µSv/procedure. The literature reports higher values for the hand between 27 and 640 µSv/procedure.^17^, ^18^ Nevertheless, the radiation dose limit for extremities (500 mSv) is unlikely to be exceeded during ERCP procedures. However, clinically this will be heavily dependent on operator work practice. If an operator is routinely subjected to procedures that require the hands to be close to the X‐ray field, the operator should consider optimization strategies such as procedural modifications or radioprotective gloves.

When using the mobile lead shield, up to 98% reduction in dose rate was measured. This did not only shield the area where it was placed but also reduced exposure to all locations beyond the shield. The location of the shield in this study was selected as it provided an area with a high dose rate and clearance from the equipment without interfering with the C‐arm. This location may not be replicated in practice if that is where the gastroenterologist is standing, as you cannot insert an endoscope behind the shield. Although this type of shield may only sometimes be possible for the gastroenterologist or to be used in that location, it should be encouraged for other staff that does not require to operate directly on the patient. The advantage of this type of shielding can be seen in Figure 3, where all locations beyond the shielded region were effectively reduced to background radiation. This creates safe zones for multiple staff to stand during exposure. Of course, the level of coverage will depend on the shield's size and proximity to the patient or source of scatter. This should be considered before recommending multiple staff standing behind a single shield.

A single apron was used to simulate the scenario of staff members using the primary shielded gastroenterologist as a barrier. As shown in Figure 4, this provides limited protection to staff if they do not align perfectly with the gastroenterologist. Therefore, if possible, staff should use other staff members as shields; however, this must not replace their protective equipment as they cannot guarantee adequate coverage.

This study presents easy‐to‐use visual guides of the ambient radiation dose in locations surrounding the fluoroscopy equipment. Although the magnitude of exposure may differ in clinical studies and other phantom studies, the relative exposure in different areas should remain similar. For example, the absolute radiation dose at the 1 m region may differ from this study; however, the relative reduction in dose at further distances or near the C‐arm should be similar. The authors recommend that these visual guides be placed around the Endoscopy Lab to inform staff which areas within the room may expose them to a higher radiation dose. This awareness may help staff members reduce their exposure to radiation during ERCP procedures. Within our setting, these visual representations, in conjunction with Figure 6, help determine which staff require what level of radiation protection.

It is important to note that the location of staff members in the procedure room can vary throughout the ERCP procedure. While the dose simulated to the gastroenterologist in our study represents the estimated dose to the primary operator, it should not be assumed to represent the dose received by other staff members. Instead, the heatmaps generated from the simulations can be used to estimate the dose received by other staff members, such as assistants and nurses, by correlating them with the typical positions these staff members occupy during the procedure. By using these heatmaps, it may be possible to develop more targeted strategies for reducing individual radiation exposure for all staff involved in ERCP procedures.

Our study has several limitations that should be considered when interpreting the results. Firstly, the data assumed that the positions of the staff remained unchanged throughout the procedure and applied a median fluoroscopy time to estimate the dose per procedure at specific locations. This may not be reflective of real‐world radiation exposure, as the heights and positions of operators may vary, and the complexity of the patients can impact the radiation exposure. Therefore, our data should be used as a guide rather than a substitute for individual radiation dose measurements. Additionally, the median fluoroscopy time used in this study to normalize the exposure per procedure was for all ERCP procedures and did not distinguish between diagnostic and therapeutic studies.

## CONCLUSION

This study indicated that the level of radiation that staff is exposed to during ERCP procedures is not trivial. Mobile shields and lead aprons can significantly reduce the staff's overall effective dose, regardless of their location in the room. For staff within 1m of the patient, radioprotective eyewear should be considered based on the results of this study and recent literature. Other staff in the room who are further from the patient are protected through distance; thus, radioprotective eyewear may not be required. The figures presented in this study can provide visual guidance to radiation exposure in areas surrounding the patient during fluoroscopic screening. This can assist in increasing awareness for the staff and providing data to determine radiation protection levels for different staff types.

## CONFLICT OF INTEREST STATEMENT

None.
